# Supplementary material for: Phosphorylated tau 181 and 217 are elevated in serum and muscle of patients with amyotrophic lateral sclerosis
Source: Nat Commun. 2025 Mar 5;16:2019. doi: 10.1038/s41467-025-57144-7 (PMC11882981; doi:10.1038/s41467-025-57144-7)
Supplement: Supplementary file 1 — Supplementary Information [file 41467_2025_57144_MOESM1_ESM.pdf]

SUPPLEMENTARY INFORMATION

SUPPLEMENTARY RESULTS

Associations between demographic variables and biomarkers

Serum p-tau 181 and serum p-tau 217 were weakly to moderately associated with age in the whole cohort (n=385, p-tau 181: Spearman's r=0.385, p<0.001, p-tau 217: r=0.471, p<0.001), in the ALS (n=152, p-tau 181: r=0.164, p=0.044; p-tau 217, r=0.161, p=0.048), and disease control (n=99, p-tau 181: r=0.430, p<0.001; p-tau 217, r=0.405, p<0.001) but not in the AD and healthy control group. In the combined group of controls (disease controls and healthy controls, n=122) age had moderate to strong associations with serum p-tau 181 (r=0.556, p<0.001) and p-tau 217 values (r=0.702, p<0.001) (Supplementary Figure 1). In the whole cohort (n=385), serum p-tau 181 and serum p-tau 217 levels were higher in males (n=205) than females (n=180, p<0.001 and p=0.006, respectively). Serum t-tau was weakly associated with age in the whole cohort (n=357, Spearman's r=0.251, p<0.001) and in the ALS group (n=141, p-tau 181: r=0.206, p=0.014), but not in the AD, disease control and healthy control groups. There was no effect of sex on serum t-tau levels.

Biomarker comparisons among diagnostic groups including healthy controls

In the whole cohort including also healthy controls (n=385), serum p-tau 181 was higher in patients with AD (n=111) and ALS (n=152) compared to disease controls (n=99, both p<0.001) and healthy controls (n=23, both p <0.001). There was no difference in serum p-tau 181 concentrations between patients with AD and ALS, whereas disease controls (n=90) had higher p-tau 181 levels compared to healthy controls (n=23, p=0.009), probably due to age effect on biomarker levels. Indeed, after age- and sex-adjustment there was no statistically significant difference between healthy controls and disease controls.

In the whole cohort, patients with AD (n=111) and ALS (n=152) showed higher serum p-tau 217 levels compared to disease controls (n=99, both p<0.001) and healthy controls (n=23, both p<0.001). Moreover,

serum p-tau 217 concentrations were more elevated in AD compared to ALS ($p<0.001$). Disease controls and healthy controls did not differ in serum p-tau 217 concentrations.

Serum t-tau was higher in AD subjects (n=111) compared to ALS cases (n=152, $p<0.001$), disease controls (n=90, $p<0.001$) and healthy controls (n=23, p=0.003), with no difference between the latter three groups.

Further analyses on serum t-tau

In the whole cohort serum t-tau was weakly to moderately associated with serum p-tau 181 (n=357, r=0.141, p=0.008), serum p-tau 217 (n=357, r=0.321, $p<0.001$), serum NfL (n=357, r=0.201, p=0.018), CSF t-tau (n=309, r=0.425, $p<0.001$), and CSF p-tau 181 (n=309, r=0.373, $p<0.001$).

In AD serum NfL and serum t-tau were correlated with serum p-tau 181 (n=111, r=0.325, p=0.001; r=0.282, p=0.004, respectively) and serum p-tau 217 (n=111, r=0.270, p=0.004; r=0.361, $p<0.001$, respectively).

In the whole analysed sample (n=357), serum t-tau showed a moderate diagnostic value in discriminating AD from ALS (AUC 0.763±0.031) or disease controls (AUC 0.756±0.035) but was not able to differentiate ALS from disease controls (0.531±0.041).

SUPPLEMENTARY METHODS

Multicentre biomarker cohort analysis

Blood and CSF biomarker analyses

The p-tau 181 and p-tau 217 Simoa assays have been commercialised for use with EDTA plasma by the manufacturer. However, previous studies have shown comparable performance and strong correlations between serum and plasma values measured with the p-tau 181 assay[1,2].

For the measurement of p-tau 217 in serum, we used the same conditions as suggested for plasma in the kit instructions, namely 73 microlitres of sample per well of a 96-well plate and a 1:3 standard dilution. All measured serum levels were above the detection limit of the assay (0.0008 pg/mL). To test the comparability of the assay in serum and plasma, we also examined correlations between plasma and serum p-tau 217 levels. From the Halle cohort, 14 participants (9 ALS, 2 AD and 4 controls) had paired serum and plasma samples

available. We measured p-tau 217 in both matrices using the Simoa assay. Median plasma p-tau 217 concentrations showed a non-significant tendency to be higher than corresponding serum values [median (IQR) plasma vs. serum 0.52 (0.26-0.98) vs. 0.29 (0.12-0.51), p=0.067]. There was a strong correlation between serum and plasma paired samples (n=14, r=0.897, p<0.001). We decided to repeat the same analyses by adding more paired serum and plasma samples collected at the Department of Neurology, Martin-Luther-University Halle-Wittenberg, Halle (Saale), resulting in a final sample size of 27 subjects. We found, here, even a stronger correlation between the paired serum and plasma samples (n=27, r=0.977, p<0.001), suggesting reliability of the p-tau 217 assay for serum samples.

We attempted to measure p-tau 217 in CSF with the same Simoa assay, but due to a matrix effect we did not obtain reliable measurements, therefore we decided not to perform p-tau 217 analyses in CSF.

Mass spectrometry-based phosphopeptide analysis of muscle biopsies

Protein digest and phosphopeptide enrichment

Protein extracts from muscle biopsies from ALS patients (5 out of the 13 cases) and disease controls (5 out of the 14 cases) (Supplementary Table 6) were subjected to proteolytic cleavage using a paramagnetic bead approach[3]. 150 µg of protein from each sample was precipitated on magnetic carboxylate-modified particles (Sigma-Aldrich, USA), reduced with 50 mM TCEP (Sigma-Aldrich, USA), carbamidomethylated with 100 mM IAA (Merck, Germany), and proteolytically cleaved with trypsin/Lys-C (Promega, USA) applied in a 1:40 trypsin/protein ratio. The peptides were subsequently collected and dried in a vacuum concentrator.

Phosphopeptides were sequentially enriched by TiO2- and Fe-NTA-based affinity chromatography as previously described[4]. Firstly, the High-Select™ TiO2 Phosphopeptide Enrichment Kit (Thermo Scientific, USA) was utilized following the instructions provided by the manufacturer. The flow-through of peptide samples and the first wash fractions were combined and dried to completeness for the following second enrichment step. The second enrichment step was conducted using the High-Select™ Fe-NTA Phosphopeptide Enrichment Kit (Thermo Scientific, USA), following the manufacturer's instructions. The phosphopeptide-enriched eluates from both steps were combined, dried to completeness, and reconstituted in 0.1% FA for LC-MS/MS measurement.

LC-MS/MS analysis

The LC-MS/MS analysis of the samples was conducted blinded to diagnoses on an EASY-nLC 1200 system (Thermo Fisher Scientific, USA), which was coupled to an Orbitrap Fusion Lumos Tribrid mass spectrometer (Thermo Fisher Scientific, USA) via a FAIMS Pro DUO interface (Thermo Fisher Scientific, USA). The peptides were trapped and desalinated on a C18 pre-column (Acclaim PepMap 100, 75 µm x 2 cm, C18) and subsequently separated on a C18 analytical column (Aurora Ultimate TS, 75 µm x 25 cm, C18).

For phosphopeptide analysis, a tripartite linear 165 min gradient with 400 nL/min flow starting from 6 % eluent B (0.1 % FA in 80 % ACN) in eluent A (0.1 % FA in water) to 45 % eluent B via 24 % eluent B after 96 min and 34 % eluent B after 140 min was used. After each sample, the column was flushed to 98% eluent B and reconstituted to starting conditions. Mass spectra were acquired in a data-dependent manner. For MS1 scans the following parameters were set: m/z range 375-1500, maximum injection time = 246 ms, normalized AGC target = 100%, R = 120 000. The most abundant ions were selected for MS2 acquisition with a fixed cycle time of 1s using the following parameters: isolation window of 1.6 m/z, maximum injection time 75 ms, normalized AGC target = 100%, normalized collision energy (NCE) = 28, R = 15 000. Fragmented ions were dynamically excluded for 60 s. The FAIMS-CV was set to alternating cycles of -50V, -65V, and -80V.

The LC-MS/MS raw data were examined by MaxQuant (Version 2.4.0.0)[5]. Database search was performed against the UniProt Homo Sapiens Reviewed RefSet (03/2024, 20418 entries + isoforms) and a list of common contaminants provided by MaxQuant (07/2019, 245 entries)[6].

Search parameters were set as follows: Maximum missed cleavages = 2, minimal peptide length = 7 amino acids, first search peptide tolerance = 20 ppm, main search peptide tolerance = 4.5 ppm, FTMS MS/MS match tolerance = 20 ppm. Carbamidomethylation of cysteine was set as fixed modification, protein N-terminal acetylation, oxidation of methionine, and phosphorylation of Serine, Threonine, and Tyrosine were set as variable modifications. Peptides, proteins, and sites were filtered by a target-decoy approach to an FDR ≤ 0.01 using a reversed decoy database. Match between runs was enabled with a match time window of 0.4 min and an alignment time window of 20 min.

Stringent filtering was applied to the phosphosites identified by our mass spectrometry approach. All sites had to pass filtering for a false discovery rate of less than 0.01. The identified phosphosites were filtered based on the phosphorylated protein. Only sites identified in the protein sequence of any tau isoform were further

analyzed. The site localization probability had to be >0.95 for singly phosphorylated peptides or between 0.475 and 0.525 for adjacent acceptor sites in doubly phosphorylated peptides. The overall posterior error probability had to be less than 0.02. In a final check, the identification score (delta score relative to the unmodified peptide) had to be at least 40 points greater than for the unphosphorylated counterpart. For proline-containing tau-phosphopeptides, all compensation voltage lines in all raw data were manually checked to exclude missing values due to proline isomerization.

Supplementary table 1. Demographic characteristics and serum biomarker distribution in healthy controls (n=23).

**Age (years) mean±SD**<br>**min-max**	35.7±7.7<br>22 – 48
**Female (%)**	65.2
**Serum p-tau 181 (pg/ml)**<br>**Median (IQR)**	0.5 (0.3-0.7)
**Serum p-tau 217 (pg/ml)**<br>**Median (IQR)**	0.10 (0.07-0.14)
**Serum t-tau (pg/ml)**<br>**Median (IQR)**	0.153 (0.076-0.223)
**Serum NfL (pg/ml)**<br>**Median (IQR)**	6.0 (3.0-10.0)

IQR interquartile range, *max* maximal value, *min* minimal value, *NfL* neurofilament light chain protein, *p-tau* phosphorylated tau protein, *SD* standard deviation, *t-tau* total tau protein

Supplementary table 2. Demographic characteristics and biomarker distribution in the diagnostic groups in the Halle cohort.

Halle				
	**ALS**	**AD**	**Disease controls**	**p-value**
**N with serum samples**	63 (38 with both serum and CSF)	66	40	
**Age (years) mean±SD**	63.9±10.7	75.7±7.7	60.3±13.9	<0.001
**Female (%)**	28.6	62.1	57.5	<0.001
**Serum p-tau 181 (pg/ml) Median (IQR)**	3.9 (1.9-5.9)	2.6 (1.7-3.6)	1.0 (0.7-1.5)	<0.001
**Serum p-tau 217 (pg/ml) Median (IQR)**	0.40 (0.25-0.54)	0.70 (0.41-0.94)	0.14 (0.10-0.18)	<0.001
**Serum t-tau (pg/ml) Median (IQR)**	0.144 (0.099-0.208)	0.308 (0.211-0.435)	0.176 (0.088-0.252)	<0.001
**Serum NfL (pg/ml) Median (IQR)**	107.0 (57.5-172.3)	47.0 (32.0-63.0)	18.1 (11.8-33.6)	<0.001
**CSF p-tau 181 (pg/ml) Median (IQR)**	31.2 (21.0-41.0)	106.7 (79.8-128.8)	26.3 (21.9-32.4)	<0.001
**CSF NfH (pg/ml) Median (IQR)**	8331 (3400-12510)	1912 (1317-2519)	1026 (710-1499)	<0.001

Age, sex and biomarkers (serum p-tau 181, serum p-tau 217, serum t-tau, serum NfL, CSF p-tau 181, CSF NfH) in the three diagnostic groups from all cohorts are displayed as mean±standard deviation (SD), median and interquartile range (IQR) or as percentage. Depending on the type and distribution of the data, two-sided p-values of ANOVA, Kruskal-Wallis or Chi-test are reported. Dunn post-hoc tests (adjustments for multiple comparisons) to compare serum t-tau, CSF p-tau 181 and neurofilament levels: serum t-tau: AD vs. controls p<0.001; AD vs. ALS p<0.001; ALS vs disease controls p=1.000; serum NfL: ALS vs AD p=0.004, ALS vs. controls p<0.001, AD vs. controls p<0.001; CSF p-tau 181: AD vs. ALS p<0.001, ALS vs. controls p=0.618, AD vs. controls p<0.001; CSF NfH: ALS vs. AD p<0.001, ALS vs. controls p<0.001, AD vs controls p=0.001. *AD* Alzheimer's disease, *ALS* amyotrophic lateral sclerosis, *CSF* cerebrospinal fluid, *IQR* interquartile range, *N* number of cases, *NfH* neurofilament heavy chain protein, *NfL* neurofilament light chain protein, *p-tau* phosphorylated tau protein, *t-tau* total tau protein

Supplementary table 3. Demographic characteristics and biomarker distribution in the diagnostic groups in the Milan cohort.

Milan				
	**ALS**	**AD**	**Disease controls**	**p-value**
**N with serum samples**	67	20	22	
**Age (years) mean±SD**	62.6±10.3	73.2±4.6	63.0±14.7	<0.001
**Female (%)**	34.3	40.0	31.8	n.s.
**Serum p-tau 181 (pg/ml) Median (IQR)**	2.3 (1.2-4.2)	3.0 (2.7-3.6)	1.2 (0.9-2.3)	0.002
**Serum p-tau 217 (pg/ml) Median (IQR)**	0.37 (0.20-0.59)	0.62 (0.40-0.86)	0.13 (0.11-0.22)	<0.001
**Serum t-tau (pg/ml) Median (IQR)**	0.144 (0.094-0.202)	0.219 (0.187-0.341)	0.148 (0.081-0.277)	<0.001
**Serum NfL (pg/ml) Median (IQR)**	127 (100.5-193.8)	39.5 (26.0-54.9)	24.7 (15.2-30.1)	<0.001
**CSF p-tau 181 (pg/ml) Median (IQR)**	28.9 (21.2-35.9)	107.9 (81.7-240.0)	29.1 (23.8-39.4)	<0.001
**CSF NfH (pg/ml) Median (IQR)**	9903 (5471-16432)	1415 (1133-1888)	1232 (994-1726)	<0.001

Age, sex and biomarkers (serum p-tau 181, serum p-tau 217, serum t-tau, serum NfL, CSF p-tau 181, CSF NfH) in the three diagnostic groups from all cohorts are displayed as mean±standard deviation (SD), median and interquartile range (IQR) or as percentage. Depending on the type and distribution of the data, two-sided p-values of ANOVA, Kruskal-Wallis or Chi-test are reported. Dunn post-hoc tests (adjustments for multiple comparisons) to compare serum t-tau, CSF p-tau 181 and neurofilament levels between diagnostic groups: serum t-tau: AD vs. controls p=0.032; AD vs. ALS p=0.002; ALS vs. disease controls p=1.000.; serum NfL: ALS vs. AD p<0.001, ALS vs. controls p<0.001, AD vs. controls p=0.029; CSF p-tau 181: AD vs. ALS p<0.001, ALS vs. controls p=1.000, AD vs. controls p<0.001; CSF NfH: ALS vs. AD p<0.001, ALS vs. controls p<0.001, AD vs. controls p=1.000 *AD* Alzheimer's disease, *ALS* amyotrophic lateral sclerosis, *CSF* cerebrospinal fluid, *IQR* interquartile range, *N* number of cases, *NfH* neurofilament heavy chain protein, *NfL* neurofilament light chain protein, *p-tau* phosphorylated tau protein, *t-tau* total tau protein

Supplementary table 4. Demographic characteristics and biomarker distribution in the diagnostic groups in the Mannheim 1 cohort.

Mannheim 1				
	**ALS**	**AD**	**Disease controls**	**p-value**
**N with serum samples**	22	-	12	
**Age (years) mean±SD**	57.0±16.2	-	61.6±17.8	n.s.
**Female (%)**	40.9	-	50.0	n.s.
**Serum p-tau 181 (pg/ml) Median (IQR)**	2.3 (1.5-3.9)	-	1.4 (1.0-2.2)	0.074
**Serum p-tau 217 (pg/ml) Median (IQR)**	0.22 (0.14-0.51)	-	0.14 (0.08-0.29)	0.043
**Serum t-tau (pg/ml) Median (IQR)**	0.043 (0.035-0.065)		0.082 (0.049-0.110)	n.s.
**Serum NfL (pg/ml) Median (IQR)**	90.8 (33.0-156.2)	-	22.9 (7.6-45.6)	0.001
**CSF p-tau 181 (pg/ml) Median (IQR)**	29.7 (18.4-44.2)	-	23.1 (18.1-47.6)	<0.001
**CSF NfH (pg/ml) Median (IQR)**	4831 (2556-9456)	-	955 (285-1361)	<0.001

Age, sex and biomarkers (serum p-tau 181, serum p-tau 217, serum NfL, CSF p-tau 181, CSF NfH) in the three diagnostic groups from all cohorts are displayed as mean±standard deviation (SD), median and interquartile range (IQR) or as percentage. Depending on the type and distribution of the data, two-sided p-values of t-test, Mann-Whitney or Chi-test are reported. Mann-Whitney tests to compare serum t-tau, CSF p-tau 181 and neurofilament levels between diagnostic groups: serum t-tau: ALS vs disease controls p=0.121.; serum NfL: ALS vs controls p<0.001; CSF p-tau 181: ALS vs controls p=0.407; CSF NfH: ALS vs controls p<0.001. *AD* Alzheimer's disease, *ALS* amyotrophic lateral sclerosis, *CSF* cerebrospinal fluid, *IQR* interquartile range, *N* number of cases, *NfH* neurofilament heavy chain protein, *NfL* neurofilament light chain protein, *p-tau* phosphorylated tau protein, *t-tau* total tau protein

Supplementary table 5. Demographic characteristics and biomarker distribution in the diagnostic groups in the Mannheim 2 cohort.

Mannheim 2				
	**ALS**	**AD**	**Disease controls**	**p-value**
**N with serum samples**	**-**	25	25	
**Age (years) mean±SD**	-	72.7±8.0	62.1±9.9	<0.001
**Female (%)**	-	56.0	64.0	n.s.
**Serum p-tau 181 (pg/ml) Median (IQR)**	-	3.1 (1.9-3.9)	1.1 (0.8-1.3)	<0.001
**Serum p-tau 217 (pg/ml) Median (IQR)**	-	0.77 (0.55-0.93)	0.15 (0.12-0.26)	<0.001
**Serum t-tau (pg/ml) Median (IQR)**		0.160 (0.110-0.218)	0.082 (0.030-0.165)	0.009
**Serum NfL (pg/ml) Median (IQR)-**	-	42.9 (38.3-60.7)	23.8 (16.7-41.1)	0.001
**CSF p-tau 181 (pg/ml) Median (IQR)**	-	132.0 (115.0-157.0)	34.3 (30.0-49.0)	<0.001
**CSF NfH (pg/ml) Median (IQR)**	-	1545 (1184-1790)	889 (757-1403)	0.001

Age, sex and biomarkers (serum p-tau 181, serum p-tau 217, serum t-tau, serum NfL, CSF p-tau 181, CSF NfH) in the three diagnostic groups from all cohorts are displayed as mean±standard deviation (SD), median and interquartile range (IQR) or as percentage. Depending on the type and distribution of the data, two-sided p-values of t-test, Mann-Whitney or Chi-test are reported. Mann-Whitney tests to compare serum t-tau, CSF p-tau 181 and neurofilament levels between diagnostic groups: serum t-tau: AD vs. controls p=0.009; serum NfL: AD vs controls p=0.002; CSF p-tau 181: AD vs controls p<0.001; CSF NfH: AD vs controls p=0.001. *AD* Alzheimer's disease, *ALS* amyotrophic lateral sclerosis, *CSF* cerebrospinal fluid, *IQR* interquartile range, *N* number of cases, *NfH* neurofilament heavy chain protein, *NfL* neurofilament light chain protein, *p-tau* phosphorylated tau protein, *t-tau* total tau protein

Supplementary Table 6. Description of ALS cases and disease controls, who underwent muscle biopsies.

ALS					
Subj. No.	***Diagnosis***	***Age onset (years)***	***Age at biopsy (years)***	***Sex***	***Muscle biopsy site and side***
1	possible ALS	62	64	F	tibialis anterior, right
2	definite ALS	68	69	M	biceps brachii, right
3	possible ALS	52	53	F	biceps brachii, right
4	definite ALS	73	75	M	vastus lateralis, right
5	possible ALS	75	76	M	Deltoideus, right
6	possible ALS	49	55	F	biceps brachii, left
7	probable ALS, laboratory supported	60	60	M	biceps brachii, left
8	definite ALS	39	40	F	vastus lateralis, left
9	possible ALS	34	35	M	biceps brachii, left
10	possible ALS, FTD	53	54	F	vastus lateralis, right
11	possible ALS	39	40	M	tibialis anterior, left
12	possible ALS	42	43	M	biceps brachii, right
13	probable ALS, laboratory supported	66	67	F	biceps brachii, left
Median		***53***	***55***		
Mean		***54,8***	***56,2***		
Minimum		***34***	***35***		
Maximum		***75***	***76***		
**Disease controls**					
Subj. No.	***Diagnosis***	***Age onset (years)***	***Age at biopsy (years)***	***Sex***	***Muscle biopsy site and side***
1	exercise intolerance	adolescence	45	M	biceps brachii, left
2	exercise interolerance, myalgia	41	46	F	biceps brachii, left
3	myalgia, cramps	51	53	M	biceps brachii, left
4	exercise interolerance	38	40	F	biceps brachii, right
5	Myalgia	28	31	M	biceps brachii, left
6	Myalgia	48	50	M	biceps brachii, left
7	myalgia, cramps	45	48	F	vastus lateralis, right
8	exercise interolerance, myalgia	43	45	M	biceps brachii, left
9	myalgia, cramps	adolescence	31	M	biceps brachii, left
10	Myalgia	49	52	M	biceps brachii, left
11	myalgia, cramps	45	49	M	biceps brachii, left
12	exercise interolerance	24	42	M	vastus lateralis, right
13	exercise interolerance, myalgia	15	20	F	Gastrocnemius, right
14	Myalgia	39	42	F	biceps brachii, left
Median		***43***	***45***		
Mean		***38,8***	***42,5***		
Minimum		***15***	***20***		
Maximum		***51***	***53***		

Immunohistochemical analysis was performed in all cases shown. Mass spectrometric analysis of muscle biopsies was performed in subjects highlighted in orange. *M* male, *F* female, *FTD* frontotemporal dementia

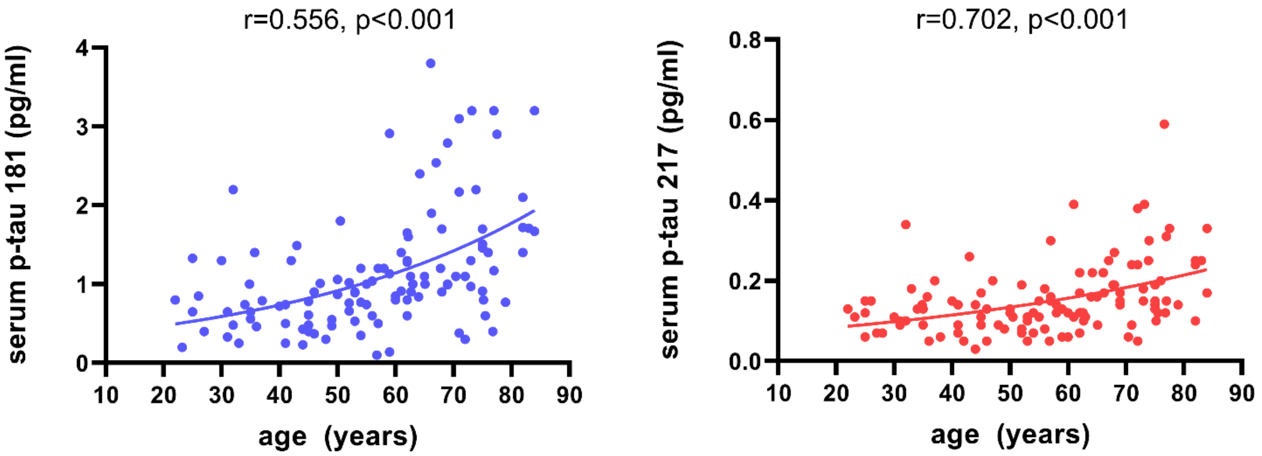

Supplementary Figure 1. Associations between age and serum p-tau 181 and serum p-tau 217 in controls. In the combined group of controls (disease controls + healthy controls, n=122) age had moderate to strong associations with serum p-tau 181 (r=0.556, p<0.001) and p-tau 217 values (r=0.702, p<0.001). Source data are provided as a Source Data file.

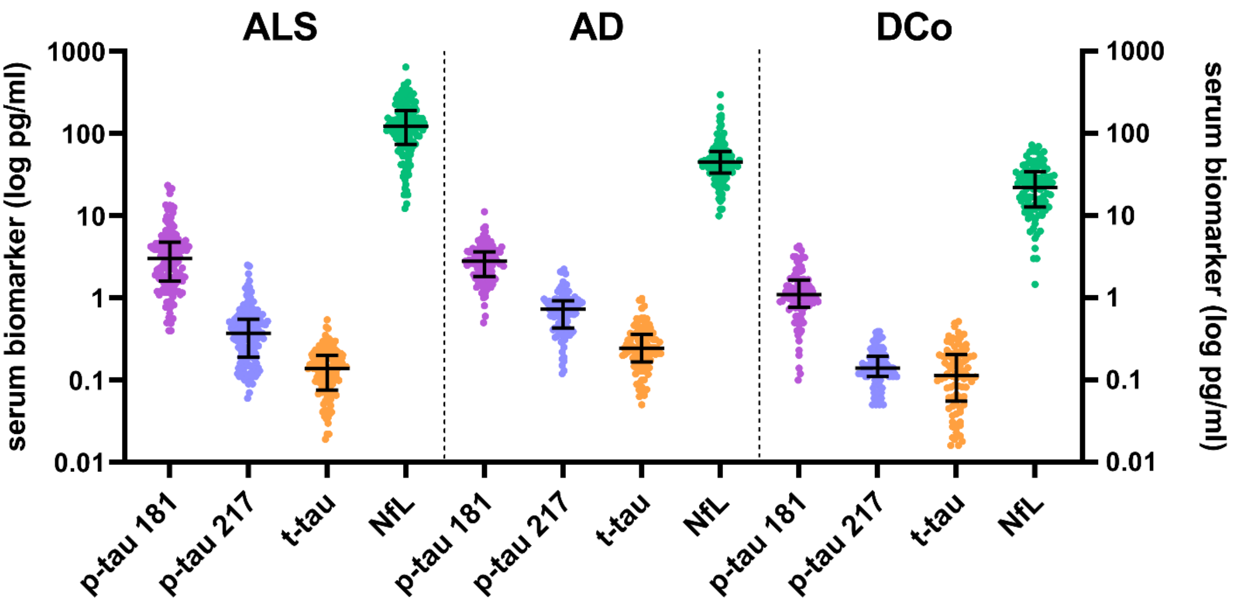

Supplementary Figure 2. Distribution of serum p-tau 181, serum p-tau 217, serum t-tau and serum NfL in the total cohort of ALS (n=152), AD cases (n=111) and disease controls (DCo, n=99).

Biomarker levels are reported on a logarithmic scale. Dots represent single data points. Horizontal lines represent the median values, the lower and upper lines correspond to the first and third quartiles, and the vertical line is the interquartile range. *AD* Alzheimer's disease, *ALS* amyotrophic lateral sclerosis, *DCo* disease controls; *NfL* neurofilament light chain protein, *p-tau* phosphorylated tau protein, *t-tau* total tau protein. Source data are provided as a Source Data file.

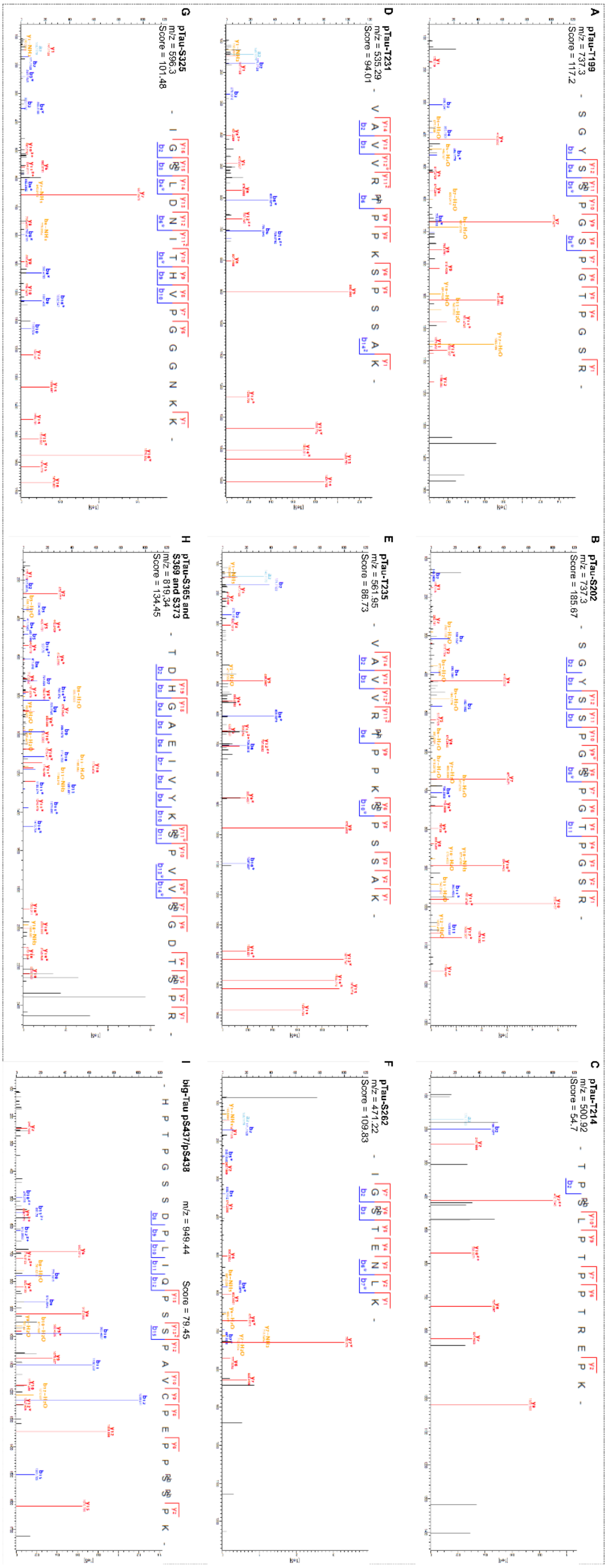

Supplementary Figure 3. Annotated spectra for further identified phosphosites in Tau. T199 (A), S202 (B), T214 (C), T231 (D), T235 (E), S262 (F), S325 (G), S365, S369, and S373 from a triply phosphorylated peptide (H), and the big-Tau exclusive phosphorylations S437 and S438 (I). Source data are provided as a Source Data file.

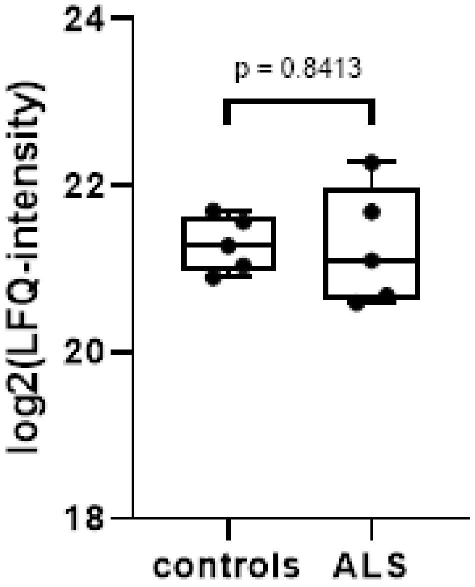

Supplementary Figure 4. Total tau abundance in muscle biopsies from ALS patients and disease controls analysed by mass spectrometry. LFQ-intensities are reported on a logarithmic scale. *LFQ* label free quantification, *ALS* amyotrophic lateral sclerosis. Source data are provided as a Source Data file.

SUPPLEMENTARY REFERENCES

1. Kac, P. R. *et al.* Diagnostic value of serum versus plasma phospho-tau for Alzheimer's disease. *Alzheimers Res Ther* **14**, 65 (2022).
2. Traub, J. *et al.* Serum phosphorylated tau protein 181 and neurofilament light chain in cognitively impaired heart failure patients. *Alzheimers Res Ther* **14**, 149 (2022).
3. Hughes, C. S. *et al.* Single-pot, solid-phase-enhanced sample preparation for proteomics experiments. *Nat Protoc* **14**, 68–85 (2019).
4. Großkopf, H., Walter, K., Karkossa, I., von Bergen, M. & Schubert, K. Non-Genomic AhR-Signaling Modulates the Immune Response in Endotoxin-Activated Macrophages After Activation by the Environmental Stressor BaP. *Front Immunol* **12**, 620270 (2021).
5. Cox, J. *et al.* Accurate proteome-wide label-free quantification by delayed normalization and maximal peptide ratio extraction, termed MaxLFQ. *Mol Cell Proteomics* **13**, 2513–26 (2014).
6. UniProt Consortium. UniProt: a worldwide hub of protein knowledge. *Nucleic Acids Res* **47**, D506–D515 (2019).
