# Supplementary material for: Phosphorylated tau 181 and 217 are elevated in serum and muscle of patients with amyotrophic lateral sclerosis
Source: Nat Commun. 2025 Mar 5;16:2019. doi: 10.1038/s41467-025-57144-7 (PMC11882981; doi:10.1038/s41467-025-57144-7)
Supplement: Supplementary file 2 — Reporting Summary [file 41467_2025_57144_MOESM2_ESM.pdf]

nature portfolio

Corresponding author(s): Prof. Markus Otto

Last updated by author(s): Feb 12, 2025

Reporting Summary

Nature Portfolio wishes to improve the reproducibility of the work that we publish. This form provides structure for consistency and transparency in reporting. For further information on Nature Portfolio policies, see our Editorial Policies and the Editorial Policy Checklist.

Statistics

For all statistical analyses, confirm that the following items are present in the figure legend, table legend, main text, or Methods section.

n/a	Confirmed	
☐	☒	The exact sample size (*n*) for each experimental group/condition, given as a discrete number and unit of measurement
☐	☒	A statement on whether measurements were taken from distinct samples or whether the same sample was measured repeatedly
☐	☒	The statistical test(s) used AND whether they are one- or two-sided<br>*Only common tests should be described solely by name; describe more complex techniques in the Methods section.*
☐	☒	A description of all covariates tested
☐	☒	A description of any assumptions or corrections, such as tests of normality and adjustment for multiple comparisons
☐	☒	A full description of the statistical parameters including central tendency (e.g. means) or other basic estimates (e.g. regression coefficient) AND variation (e.g. standard deviation) or associated estimates of uncertainty (e.g. confidence intervals)
☐	☒	For null hypothesis testing, the test statistic (e.g. *F*, *t*, *r*) with confidence intervals, effect sizes, degrees of freedom and *P* value noted<br>*Give P values as exact values whenever suitable.*
☒	☐	For Bayesian analysis, information on the choice of priors and Markov chain Monte Carlo settings
☒	☐	For hierarchical and complex designs, identification of the appropriate level for tests and full reporting of outcomes
☐	☒	Estimates of effect sizes (e.g. Cohen's *d*, Pearson's *r*), indicating how they were calculated

Our web collection on statistics for biologists contains articles on many of the points above.

Software and code

Policy information about availability of computer code

Data collection	We collected data in Excel (Microsoft)
Data analysis	We used IBM SPSS Statistics V.21 (IBM), GraphPad Prism V.7 (GraphPad Software, La Jolla, California, USA), and R software V.4.0.2 (R foundation, Vienna, Austria).

For manuscripts utilizing custom algorithms or software that are central to the research but not yet described in published literature, software must be made available to editors and reviewers. We strongly encourage code deposition in a community repository (e.g. GitHub). See the Nature Portfolio guidelines for submitting code & software for further information.

Data

Policy information about availability of data

All manuscripts must include a data availability statement. This statement should provide the following information, where applicable:

- Accession codes, unique identifiers, or web links for publicly available datasets
- A description of any restrictions on data availability
- For clinical datasets or third party data, please ensure that the statement adheres to our policy

Biomarker and mass spectrometry data generated in this study are provided in the Source Data file. Source data are provided with this paper. Mass spectrometry data have also been deposited in the ProteomeXchange Consortium database via the PRIDE partner repository under the identifiers PXD055632 [http://www.ebi.ac.uk/pride/archive/projects/PXD055632/] and PXD060425 [http://www.ebi.ac.uk/pride/archive/projects/PXD060425] for phosphopeptide analysis and protein data, respectively. All other data contain patient-related clinical information and are available on request from the corresponding author. The request will

be reviewed by the investigators and the respective institutions to verify that the data transfer is in accordance with EU legislation on general data protection or is subject to intellectual property or confidentiality obligations.

Research involving human participants, their data, or biological material

Policy information about studies with human participants or human data. See also policy information about sex, gender (identity/presentation), and sexual orientation and race, ethnicity and racism.

Reporting on sex and gender	data on sex of participants were provided from each centre.
Reporting on race, ethnicity, or other socially relevant groupings	these variables were not collected and not analysed
Population characteristics	For all ALS, AD cases and controls we collected data on age and sex. In ALS patients from Halle and Milan (n=68), we collected, when available, clinic-demographical data and scores and laboratory results: age at blood sampling, sex, body mass index (BMI), disease duration at blood sampling (time between symptom onset and blood sampling), site of symptom onset26, clinical phenotype, King's stage at blood sampling, ALS Functional Rating Scale-Revised (ALSFRS-R) score and disease progression rate at blood sampling, Edinburgh Cognitive and Behavioural ALS Screen (ECAS) total score at blood sampling, blood levels of creatine kinase (CK), troponin T, myoglobin. Clinical phenotypes comprehended classic, bulbar, upper motor neuron (UMN)-predominant (including also primary lateral sclerosis) and LMN-predominant (including flail arm syndrome, flail leg syndrome and progressive muscular atrophy) ALS. The disease progression rate at blood sampling was calculated as follows: (48−ALSFRS-R score at the time of sampling)/time between symptom onset and blood sampling.
Recruitment	In this retrospective multicentre cohort study, we included ALS and AD (dementia or mild cognitive impairment) cases and non-neurodegenerative disease controls from four different cohorts (total n=362). The case selection was based on the availability of serum samples from subjects with the target conditions and complete information for diagnostic criteria application. Participants were recruited at the Department of Neurology, Martin-Luther-University Halle-Wittenberg, Halle (Saale), Germany (Halle cohort, n=169), Department of Neurology and Laboratory of Neuroscience, IRCCS Istituto Auxologico Italiano, Milan, Italy (Milan cohort, n=109), at the Division for Neurodegenerative Diseases, Neurology Department, Mannheim Center for Translational Medicine, University Medicine Mannheim, Mannheim, Germany (Mannheim cohort 1, n=34), or at the Department of Geriatric Psychiatry, Central Institute of Mental Health, Mannheim, Germany (Mannheim cohort 2, n=50). The Halle cohort comprised 63 ALS, 66 AD and 40 disease controls, the Milan cohort 67 ALS, 20 AD and 22 disease controls, the Mannheim 1 cohort 1 22 ALS and 12 disease controls, the Mannheim 2 cohort 25 AD and 25 controls. All AD and control participants as well as 127 of 152 ALS cases had available CSF samples.<br>ALS diagnosis was made according to the Revised El Escorial criteria. The diagnosis of AD was supported by the analysis of CSF core biomarkers, according to the National Institute on Aging and Alzheimer's Association recommendations. Non-neurodegenerative disease controls comprised subjects lacking any clinical, neuroradiological and CSF evidence of neurodegenerative disease.<br>To further investigate the potential impact of age on biomarkers, we included also serum samples from 23 healthy controls aged less than 50 years with normal Mini-Mental State Examination scores, no history of neurological disease and normal serum NfL levels according to our in-house cut-off values. No CSF AD core biomarker assessment was performed in these cases, but given their age, the occurrence of incidental AD co-pathology should be considered unlikely.
Ethics oversight	The study was conducted according to the revised Declaration of Helsinki and Good Clinical Practice guidelines. Informed written consent was obtained from participants and/or their relatives. The study of biosamples and case data was approved by the ethics committees of the Martin-Luther-University Halle-Wittenberg (approval number 2021-101), IRCCS Istituto Auxologico Italiano (approval number 2023_03_21_18) and University Medicine Mannheim (approval numbers 2017-589N-MA and 2012-254N-MA). The study followed the STROBE statement guidelines.

Note that full information on the approval of the study protocol must also be provided in the manuscript.

Field-specific reporting

Please select the one below that is the best fit for your research. If you are not sure, read the appropriate sections before making your selection.

☒ Life sciences ☐ Behavioural & social sciences ☐ Ecological, evolutionary & environmental sciences

For a reference copy of the document with all sections, see nature.com/documents/nr-reporting-summary-flat.pdf

Life sciences study design

All studies must disclose on these points even when the disclosure is negative.

Sample size	No statistical method was used to predetermine sample size. The study included 385 cases/samples from four reference centres. To adress the low case number in each diagnostic group, we performed a multicentre cohort analysis.
Data exclusions	We attempted to measure p-tau 217 in CSF with the same Simoa kit, but due to a matrix effect we did not obtain reliable measurements, therefore we decided not to perform p-tau 217 analyses in CSF. No other data have been excluded.
Replication	For all biomarker measurements, the coefficients of intra-assay and inter-assay variability were <10% and <15%, respectively.

Randomization	Allocation of samples in the ELISA plates was random, also the IHC and mass spectrometric analysis of samples was random.
Blinding	All biomarker analyses were measured blind to allocation and diagnose. All IHC and mass spectrometric analyses were performed blind to allocation and diagnose.

Reporting for specific materials, systems and methods

We require information from authors about some types of materials, experimental systems and methods used in many studies. Here, indicate whether each material, system or method listed is relevant to your study. If you are not sure if a list item applies to your research, read the appropriate section before selecting a response.

Materials & experimental systems

n/a	Involved in the study
☐	☒ Antibodies
☐	☐ Eukaryotic cell lines
☐	☐ Palaeontology and archaeology
☐	☐ Animals and other organisms
☐	☒ Clinical data
☐	☐ Dual use research of concern
☐	☐ Plants

Methods

n/a	Involved in the study
☐	☐ ChIP-seq
☐	☐ Flow cytometry
☐	☐ MRI-based neuroimaging

Antibodies

Antibodies used	We used primary antibodies against p-tau 181 (Thermo Fisher Scientific, MA, USA; AT270 #MN1050; 1:50) and p-tau 217 (Thermo Fisher Scientific, MA, USA; #44–744; 1:50).<br>https://www.thermofisher.com/antibody/product/Phospho-Tau-Thr217-Antibody-Polyclonal/44-744<br>https://www.thermofisher.com/antibody/product/Phospho-Tau-Thr181-Antibody-clone-AT270-Monoclonal/MN1050
Validation	The above mentioned antibodies have been previously used in the vast majority of other publications on IHC (Kim et al. Acta neuropathologica 2023; Saunders et al. Brain communications 2023; Ercan et al. Molecular neurodegeneration 2017; Saroja et al. Alzheimers Dementia 2022). Moreover, vwe performed validation of these antibodies on brain tissue samples. Samples from the left and right motor cortex of one ALS patient were used as a reference (n=2). Here we used the same dilution and the same conditions applied for muscle tissue samples.<br>Furthermore, in order to exclude the possibility of non-specific binding of the p-tau antibodies used, we decided to also study the presence of p-tau peptides in muscle biopsies by mass spectrometry, a highly specific and reliable method. Here we confirmed the presence of p-tau 181 and p-tau 217 peptides in ALS and control muscle tissue.<br>To support the accuracy of our mass spectrometry results, the respective quality values of the measurements for all phosphorylated tau sites are included in the manuscript.

Eukaryotic cell lines

Policy information about cell lines and Sex and Gender in Research

Cell line source(s)	*State the source of each cell line used and the sex of all primary cell lines and cells derived from human participants or vertebrate models.*
Authentication	*Describe the authentication procedures for each cell line used OR declare that none of the cell lines used were authenticated.*
Mycoplasma contamination	*Confirm that all cell lines tested negative for mycoplasma contamination OR describe the results of the testing for mycoplasma contamination OR declare that the cell lines were not tested for mycoplasma contamination.*
Commonly misidentified lines<br>(See ICLAC register)	*Name any commonly misidentified cell lines used in the study and provide a rationale for their use.*

Palaeontology and Archaeology

Specimen provenance	*Provide provenance information for specimens and describe permits that were obtained for the work (including the name of the issuing authority, the date of issue, and any identifying information). Permits should encompass collection and, where applicable, export.*
Specimen deposition	*Indicate where the specimens have been deposited to permit free access by other researchers.*
Dating methods	*If new dates are provided, describe how they were obtained (e.g. collection, storage, sample pretreatment and measurement), where they were obtained (i.e. lab name), the calibration program and the protocol for quality assurance OR state that no new dates are*

provided.

☐ Tick this box to confirm that the raw and calibrated dates are available in the paper or in Supplementary Information.

Ethics oversight	*Identify the organization(s) that approved or provided guidance on the study protocol, OR state that no ethical approval or guidance was required and explain why not.*

Note that full information on the approval of the study protocol must also be provided in the manuscript.

Animals and other research organisms

Policy information about studies involving animals; ARRIVE guidelines recommended for reporting animal research, and Sex and Gender in Research

Laboratory animals	*For laboratory animals, report species, strain and age OR state that the study did not involve laboratory animals.*
Wild animals	*Provide details on animals observed in or captured in the field; report species and age where possible. Describe how animals were caught and transported and what happened to captive animals after the study (if killed, explain why and describe method; if released, say where and when) OR state that the study did not involve wild animals.*
Reporting on sex	*Indicate if findings apply to only one sex; describe whether sex was considered in study design, methods used for assigning sex. Provide data disaggregated for sex where this information has been collected in the source data as appropriate; provide overall numbers in this Reporting Summary. Please state if this information has not been collected. Report sex-based analyses where performed, justify reasons for lack of sex-based analysis.*
Field-collected samples	*For laboratory work with field-collected samples, describe all relevant parameters such as housing, maintenance, temperature, photoperiod and end-of-experiment protocol OR state that the study did not involve samples collected from the field.*
Ethics oversight	*Identify the organization(s) that approved or provided guidance on the study protocol, OR state that no ethical approval or guidance was required and explain why not.*

Note that full information on the approval of the study protocol must also be provided in the manuscript.

Clinical data

Policy information about clinical studies

All manuscripts should comply with the ICMJEguidelines for publication of clinical research and a completedCONSORT checklist must be included with all submissions.

Clinical trial registration	our study is not a clinical trial
Study protocol	our study is not a clinical trial
Data collection	our study is not a clinical trial
Outcomes	our study is not a clinical trial

Dual use research of concern

Policy information about dual use research of concern

Hazards

Could the accidental, deliberate or reckless misuse of agents or technologies generated in the work, or the application of information presented in the manuscript, pose a threat to:

No	Yes	
☐	☐	Public health
☐	☐	National security
☐	☐	Crops and/or livestock
☐	☐	Ecosystems
☐	☐	Any other significant area

Experiments of concern

Does the work involve any of these experiments of concern:

No	Yes	
☐	☐	Demonstrate how to render a vaccine ineffective
☐	☐	Confer resistance to therapeutically useful antibiotics or antiviral agents
☐	☐	Enhance the virulence of a pathogen or render a nonpathogen virulent
☐	☐	Increase transmissibility of a pathogen
☐	☐	Alter the host range of a pathogen
☐	☐	Enable evasion of diagnostic/detection modalities
☐	☐	Enable the weaponization of a biological agent or toxin
☐	☐	Any other potentially harmful combination of experiments and agents

Plants

Seed stocks	*Report on the source of all seed stocks or other plant material used. If applicable, state the seed stock centre and catalogue number. If plant specimens were collected from the field, describe the collection location, date and sampling procedures.*
Novel plant genotypes	*Describe the methods by which all novel plant genotypes were produced. This includes those generated by transgenic approaches, gene editing, chemical/radiation-based mutagenesis and hybridization. For transgenic lines, describe the transformation method, the number of independent lines analyzed and the generation upon which experiments were performed. For gene-edited lines, describe the editor used, the endogenous sequence targeted for editing, the targeting guide RNA sequence (if applicable) and how the editor was applied.*
Authentication	*Describe any authentication procedures for each seed stock used or novel genotype generated. Describe any experiments used to assess the effect of a mutation and, where applicable, how potential secondary effects (e.g. second site T-DNA insertions, mosiacism, off-target gene editing) were examined.*

ChIP-seq

Data deposition

☐ Confirm that both raw and final processed data have been deposited in a public database such as GEO.

☐ Confirm that you have deposited or provided access to graph files (e.g. BED files) for the called peaks.

Data access links<br>*May remain private before publication.*	*For "Initial submission" or "Revised version" documents, provide reviewer access links. For your "Final submission" document, provide a link to the deposited data.*
Files in database submission	*Provide a list of all files available in the database submission.*
Genome browser session<br>(e.g. UCSC)	*Provide a link to an anonymized genome browser session for "Initial submission" and "Revised version" documents only, to enable peer review. Write "no longer applicable" for "Final submission" documents.*

Methodology

Replicates	*Describe the experimental replicates, specifying number, type and replicate agreement.*
Sequencing depth	*Describe the sequencing depth for each experiment, providing the total number of reads, uniquely mapped reads, length of reads and whether they were paired- or single-end.*
Antibodies	*Describe the antibodies used for the ChIP-seq experiments; as applicable, provide supplier name, catalog number, clone name, and lot number.*
Peak calling parameters	*Specify the command line program and parameters used for read mapping and peak calling, including the ChIP, control and index files used.*
Data quality	*Describe the methods used to ensure data quality in full detail, including how many peaks are at FDR 5% and above 5-fold enrichment.*
Software	*Describe the software used to collect and analyze the ChIP-seq data. For custom code that has been deposited into a community repository, provide accession details.*

Flow Cytometry

Plots

Confirm that:

- [ ] The axis labels state the marker and fluorochrome used (e.g. CD4-FITC).
- [ ] The axis scales are clearly visible. Include numbers along axes only for bottom left plot of group (a 'group' is an analysis of identical markers).
- [ ] All plots are contour plots with outliers or pseudocolor plots.
- [ ] A numerical value for number of cells or percentage (with statistics) is provided.

Methodology

Sample preparation	*Describe the sample preparation, detailing the biological source of the cells and any tissue processing steps used.*
Instrument	*Identify the instrument used for data collection, specifying make and model number.*
Software	*Describe the software used to collect and analyze the flow cytometry data. For custom code that has been deposited into a community repository, provide accession details.*
Cell population abundance	*Describe the abundance of the relevant cell populations within post-sort fractions, providing details on the purity of the samples and how it was determined.*
Gating strategy	*Describe the gating strategy used for all relevant experiments, specifying the preliminary FSC/SSC gates of the starting cell population, indicating where boundaries between "positive" and "negative" staining cell populations are defined.*

- [ ] Tick this box to confirm that a figure exemplifying the gating strategy is provided in the Supplementary Information.

Magnetic resonance imaging

Experimental design

Design type	*Indicate task or resting state; event-related or block design.*
Design specifications	*Specify the number of blocks, trials or experimental units per session and/or subject, and specify the length of each trial or block (if trials are blocked) and interval between trials.*
Behavioral performance measures	*State number and/or type of variables recorded (e.g. correct button press, response time) and what statistics were used to establish that the subjects were performing the task as expected (e.g. mean, range, and/or standard deviation across subjects).*

Acquisition

Imaging type(s)	*Specify: functional, structural, diffusion, perfusion.*
Field strength	*Specify in Tesla*
Sequence & imaging parameters	*Specify the pulse sequence type (gradient echo, spin echo, etc.), imaging type (EPI, spiral, etc.), field of view, matrix size, slice thickness, orientation and TE/TR/flip angle.*
Area of acquisition	*State whether a whole brain scan was used OR define the area of acquisition, describing how the region was determined.*

Diffusion MRI ☐ Used ☐ Not used

Preprocessing

Preprocessing software	*Provide detail on software version and revision number and on specific parameters (model/functions, brain extraction, segmentation, smoothing kernel size, etc.).*
Normalization	*If data were normalized/standardized, describe the approach(es): specify linear or non-linear and define image types used for transformation OR indicate that data were not normalized and explain rationale for lack of normalization.*
Normalization template	*Describe the template used for normalization/transformation, specifying subject space or group standardized space (e.g. original Talairach, MNI305, ICBM152) OR indicate that the data were not normalized.*
Noise and artifact removal	*Describe your procedure(s) for artifact and structured noise removal, specifying motion parameters, tissue signals and physiological signals (heart rate, respiration).*

Volume censoring | *Define your software and/or method and criteria for volume censoring, and state the extent of such censoring.*

Statistical modeling & inference

Model type and settings | *Specify type (mass univariate, multivariate, RSA, predictive, etc.) and describe essential details of the model at the first and second levels (e.g. fixed, random or mixed effects; drift or auto-correlation).*

Effect(s) tested | *Define precise effect in terms of the task or stimulus conditions instead of psychological concepts and indicate whether ANOVA or factorial designs were used.*

Specify type of analysis: ☐ Whole brain ☐ ROI-based ☐ Both

Statistic type for inference | *Specify voxel-wise or cluster-wise and report all relevant parameters for cluster-wise methods.*

(See Eklund et al. 2016)

Correction | *Describe the type of correction and how it is obtained for multiple comparisons (e.g. FWE, FDR, permutation or Monte Carlo).*

Models & analysis

n/a	Involved in the study
☐	☐ Functional and/or effective connectivity
☐	☐ Graph analysis
☐	☐ Multivariate modeling or predictive analysis

Functional and/or effective connectivity | *Report the measures of dependence used and the model details (e.g. Pearson correlation, partial correlation, mutual information).*

Graph analysis | *Report the dependent variable and connectivity measure, specifying weighted graph or binarized graph, subject- or group-level, and the global and/or node summaries used (e.g. clustering coefficient, efficiency, etc.).*

Multivariate modeling and predictive analysis | *Specify independent variables, features extraction and dimension reduction, model, training and evaluation metrics.*
